# Hunter–Schreger Bands: Clinical Implications and Future Directions, Scoping Review

**DOI:** 10.1155/ijod/2104735

**Published:** 2025-10-14

**Authors:** Mario Dioguardi, Ciro Guerra, Gaetano Illuzzi, Lorenzo Sanesi, Diego Sovereto, Andrea Ballini, Angela Pia Cazzolla, Angelo Martella, Lorenzo Lo Muzio, Khrystyna Zhurakivska, Angela Tisci, Giuseppe Troiano

**Affiliations:** ^1^Department of Clinical and Experimental Medicine, University of Foggia, Via Rovelli 50 71122, Foggia, Italy; ^2^DataLab, Department of Engineering for Innovation, University of Salento, Lecce, Italy; ^3^Department of Medicine and Surgery, University LUM ‘Giuseppe Degennaro', Casamassima, Bari, Italy

**Keywords:** dental, enamel, enamel microstructure, enamel morphology, enamel strength, Hunter–Schreger, NCCL, tooth

## Abstract

This scoping review aims to examine and identify the potential clinical implications and future research directions concerning Hunter–Schreger bands (HSBs) through an analysis of the scientific medical literature. The scoping review was conducted following the PRISMA Extension for Scoping Reviews (PRISMA-ScR) guidelines, utilising electronic databases such as PubMed, Scopus, the Cochrane Central Register of Trials and ScienceDirect, employing keyword combinations for database search. During the selection process, 327 records were identified, of which only 10 studies fully met the inclusion criteria and were included in the review. The extracted data were summarised in tables. Current evidence suggests that HSB play a crucial role in strengthening enamel by dissipating energy and preventing crack propagation. The use of imaging technologies, optical microscopy and diffraction studies has enabled a detailed characterisation of their structure, distribution and function, opening new avenues for future diagnostic and therapeutic applications in dentistry. However, the predominance of in vitro studies highlights the need for further in vivo clinical research to confirm and translate these findings into innovative dental practices.

## 1. Introduction

**Figure 1:**
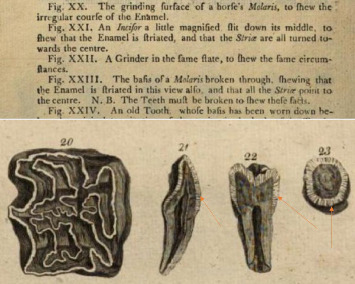
The images depicting HSBs as shown in John Hunter's book, ‘The Natural History of the Human Teeth'. The orange arrows indicate the HSBs referred to by Hunter as ‘Striae', as can be seen from the explanation provided in Figures XXI and XXIII.

Hunter–Schreger bands (HSBs) represent one of the most fascinating and complex aspects of the microstructural histology of teeth, particularly enamel. HSBs are optical phenomena observed in the enamel of mammalian teeth, particularly in species with high-wear dentition. These structures consist of alternating light and dark bands that result from the decussation (crossing) of enamel prisms at different angles. The analysis of their discovery, historical evolution, and biomechanical properties not only provides insight into the evolutionary past of living organisms but also offers valuable perspectives for diagnostic and therapeutic innovations in dentistry. The first observations of HSBs date back to 1771 with Hunter [1, 2] (Figure 1) and later in 1800 by Schreger [3]. However, the earliest notations regarding the presence of these bands probably date back to 1699, made by Gabriel-Philippe de la Hire [4], who observed them through a low-power optical microscope [5] (Figure 1).

Dental hard tissues have been widely studied in the field of evolution and anthropology thanks to their easy availability in archaeological and palaeontological sites, thanks to their high mineralisation and density. Some structural features of enamel, including HSBs, are species specific and follow evolutionary adaptation. The complexity of some of these structures such as the HSB represents the expression of evolutionary biomechanical adaptation to the management of occlusal forces [6].

HSBs are found in the enamel of most mammals [7], presenting a highly variable configuration among different species [8]. For instance, in rhinoceros molars, they are oriented vertically [9], while in calicotheres and brontotheres, they take on a U-shaped form [10]. In cave bears and polar bears, enamel consists of two layers: an inner layer composed of HSBs and an outer radial layer, with a predominance of open prisms [11].

The presence of HSBs is documented in nearly all large mammals, generally exceeding 1–2 kg [12, 13], except for rodents, which exhibit unique characteristics in their incisors [14]. Only sporadically have they also been found in some reptile species [15].

### 1.1. Origin and Physical Properties of HSB

**Figure 2:**
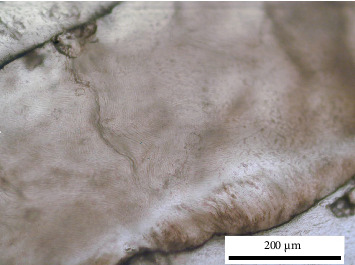
Histological section showing the different inclination of the enamel prisms. Polished ground section of human enamel imaged in reflected light (cross-polarised). Scale bar: 200 µm (estimated). Pixel size was imposed by assigning the diameter of a single enamel prism to 5 µm and measuring the median pixel length of three marked prism-width segments (Methods, ‘Scale calibration and uncertainty').

**Figure 3:**
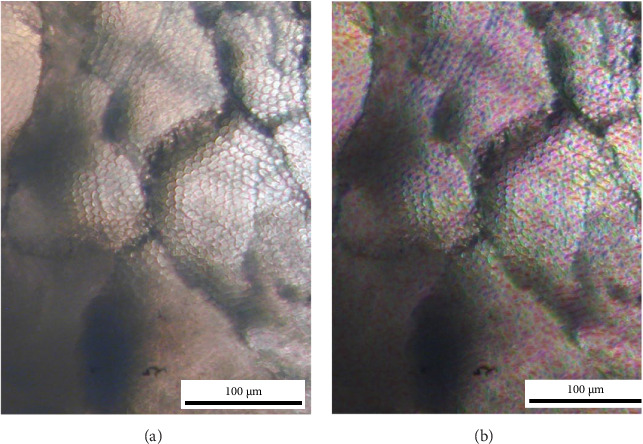
Enamel prisms and orientation map. (A) Reflected-light micrograph showing prism heads (‘honeycomb' pattern). (B) Same field with a structure-tensor orientation map overlaid (hue = orientation; saturation = coherence), providing an indirect (proxy) measure of local prism-bundle orientation rather than a direct crystallographic determination. Scale bar: 100 µm (estimated from five prism-diameter marks; Methods). The structure-tensor map is an optical, image-gradient-based crystallographic proxy of prism-bundle orientation and is not a substitute for SEM/EBSD or transmitted-light on thin sections. Limitations: results depend on image contrast/optical quality and on the assumption that the reflected texture follows prism orientation; the method does not provide lattice or chemical parameters and cannot resolve crystalline micro-defects.

Enamel prisms originate mostly from the dentino-enamel junction (DEJ) and, to a lesser extent, from the cemento-enamel junction (CEJ) and extend towards the outer surface of the enamel, where they tend to be larger. However, the outermost layer of enamel may be devoid of prisms. These prisms are composed of a mineral called hydroxyapatite, deposited by ameloblasts, which forms crystalline formations in the shape of rods known as ‘prisms', with a diameter of approximately 5 µm (Figures 2 and 3). During the mineralisation process of the enamel matrix, various hydroxyapatite crystals are formed and interconnected by thin nanometric organic layers. Inside the prisms, the crystals are not co-oriented but change orientation between 30° and 90°. This contributes to dissipating energy and imparting toughness to the mineral structure. Furthermore, the presence of organic sheaths surrounding the prisms, with differences in nanohardness and elasticity, further promotes the reduction of shear stress and transverse loads [16].

The orientation of prisms can be irregular, thus defining the enamel type. Enamel prisms typically follow a path with irregular deviations resembling waves, resulting in an increased path length compared to a straight line through the enamel thickness, by about 7% [17]. This simultaneous change in the path followed by prisms is known as the ‘prism decussation phenomenon', which creates groups of adjacent prisms with variably inclined long axes (~40°), and these characteristics underlie the optical properties of HSB.

Usually, enamel prisms follow a straight path near the CEJ before deviating further [18]. The outermost quarter of the external enamel lacks deviations, while the outermost layer consists of aprismatic enamel. In the cuspal region, prisms tend to have a nodular structure, whereas in the cervical zone, there are poorly defined prisms that follow a straight path.

The visual phenomenon observed in HSBs is not identifiable when observing individual hydroxyapatite crystals but becomes evident when the crystals combine to form prisms, and the prism bodies are oriented in parallel. The cross-section then appears dark (referred to as ‘diazone') when light strikes in a direction coinciding with the long axis of the prisms, disappearing into the depth of the enamel. On the other hand, the section appears light (referred to as ‘parazone') when light falls perpendicularly or at an angle to the prisms. The variation in prism inclination between the diazone and parazone is about 40° [19].

The visual appearance of the bands also depends on the location of the light source [19]. Variations in the inclination of the light source can cause a band to appear light first and then dark. The thickness of the band is determined by the number of prisms present, with significant variability among mammal species, ranging from a single prism for smaller mammals (mouse) [20] to 6–16 prisms for larger mammals [21].

The twisted and laminated structure of HSBs provides resistance to fracture and, thanks to the anisotropy of their structure, dissipates the energy that could generate a crack. Cracks propagate by splitting the protein-rich prismatic sheaths instead of propagating through the prismatic lattice. Diazone and parazone alterations in the prism axes aim to increase the energy required for crack propagation, which occurs when it preferably extends along the prismatic sheaths or encounters a change in prism inclination [22].

In conclusion, this preliminary overview highlights how the study of HSB serves as a crucial bridge between evolutionary research and clinical applications. This integration of knowledge paves the way for a more targeted and innovative approach in dental practice, fostering advancements in both the diagnosis and treatment of dental pathologies [23].

Therefore, the objective of this scoping review is to summarise the clinical implications and future directions of research conducted on HSB in recent years

## 2. Materials and Methods

### 2.1. Protocol and Registration

This scoping review was conducted in accordance with the PRISMA Extension for Scoping Reviews (PRISMA-ScR) checklist, as outlined by Tricco et al. [24]. Although the protocol was prepared prior to initiating the database search, it was chosen not to register it. This decision, unanimously agreed upon by all reviewers, was made because the International Prospective Register of Systematic Reviews (PROSPERO) does not allow the registration of scoping reviews.

### 2.2. Eligibility Criteria

All studies analysing HSBs and their clinical implications, particularly in dentistry, published in the last 15 years were considered potentially eligible. Over the past decade, significant advancements in imaging technologies, artificial intelligence, analytical methodologies and clinical knowledge in dentistry have provided a more in-depth and updated understanding of HSB and their clinical relevance. This timeframe allows the inclusion of studies that reflect current practices and innovations, ensuring a review that is both relevant and applicable to the modern clinical context.

Therefore, publication year restrictions were applied. However, no language restrictions were imposed. Literature reviews were excluded from the primary analysis and were used exclusively to enhance the bibliographic research and deepen the thematic understanding of the review.

### 2.3. Information Sources

The search was conducted using three major databases—PubMed, Scopus and ScienceDirect—in addition to a registry, namely the Cochrane Library. Furthermore, a grey literature search was integrated through platforms such as Google Scholar and OpenGrey (Data Archiving and Networked Services [DANS]).

This choice was motivated by the need to minimise publication bias: studies that do not report statistically significant differences between different methodologies may be excluded from publication in high-impact journals, making them less visible in traditional databases [25]. However, such data may emerge from conference proceedings, preprints or doctoral theses, representing crucial information to avoid distortions in aggregated meta-analysis results, should such an analysis be conducted in the future [26].

Restricting the selection exclusively to published studies with significant results, while excluding conference reports or papers with non-statistically significant findings, could introduce bias, potentially skewing the overall effect towards a particular treatment over the control. Although this is a scoping review, given the well-defined review objective, it was deemed necessary to expand the database search to include grey literature.

Additionally, references cited in literature reviews on HSB were examined to identify further potentially relevant articles. It should be noted that systematic and narrative reviews were excluded from the final selection, as the analysis focused solely on primary research studies.

The entire search process was conducted between 10 and 20 February 2025, with the final update of the identified records completed on 7 March 2025.

### 2.4. Search

The authors responsible for researching the studies used the following keywords in the databases, the strings used for PubMed were as follows:

Search: Hunter–Schreger bands Filters: from 2010 to 2025 Sort by: Most Recent

(“Hunter-Schreger”[All Fields] AND (“band s”[All Fields] OR “bands”[All Fields])) AND (2010:2025[pdat])

Translations

bands: “band's”[All Fields] OR “bands”[All Fields]

On Scopus: TITLE-ABS-KEY (hunter–schreger AND bands)

On Cochrane Central Register of Controlled Trials: Hunter–Schreger bands in Title Abstract Keyword.

### 2.5. Selection of Sources of Evidence

The selection of relevant articles and reports was conducted by two reviewers (Mario Dioguardi and Ciro Guerra), while a third reviewer (Diego Sovereto) responsible for resolving any discrepancies regarding study inclusion. The reviewers initially agreed on the selection criteria, the keywords to be used, and the databases to be consulted. They then carried out the search independently, recording the number of articles retrieved for each keyword and database.

Duplicates obtained from different sources were removed using EndNote 9 software, while additional duplicates that could not be automatically managed were manually eliminated during the screening phase. Subsequently, the two reviewers performed the screening and proceeded with the final selection of studies, discussing and comparing findings to determine which studies should be included.

### 2.6. Data Charting Process, Data Items and Synthesis of Results

After study selection, the two reviewers collaborated to clearly define the characteristics and information to be extracted from each study. Specifically, the collected data included: the first author's name, year of publication, bibliographic reference, study type, number of samples, examined teeth or patients and main findings or conclusions.

Across the included studies, enamel microstructure and HSB were evaluated using scanning electron microscopy (SEM), polarised-light/polarisation microscopy, optical coherence tomography (OCT), micro-computed tomography and other optical/image-processing approaches. Details for each article are summarised in the results tables.  Figure 2 shows a polished ground section imaged in reflected light (cross-polarised) to illustrate the optical appearance of HSB.

Each reviewer independently extracted the data and compiled the information into separate tables. Subsequently, a third reviewer cross-checked the data to verify the accuracy of the entries [27]. Scoping reviews typically do not include a risk of bias assessment, as a fundamental difference between scoping reviews and systematic reviews is that the former are generally conducted to provide an overview of existing evidence, regardless of methodological quality or bias risk. Therefore, while the sources of evidence included in this scoping review were critically appraised [24], only a risk of bias assessment was performed, without further discussion of its implications. For this evaluation, the Checklist for Reporting In Vitro Studies (CRISs) [28, 29] was used.

### 2.7. Additional Information: Scale Calibration (Figure 2) and Software

Because the original image lacked in-camera calibration (Figure 2), pixel size was imposed using a known morphological reference. Three short line segments were drawn across individual enamel prisms; their pixel lengths were measured and the median value (*L* ≈ 8.06 px) was used to compute the pixel size as µm/px = *d*/*L*, where *d* is the assumed prism diameter (5 µm for human enamel). Given that the drawn lines are very short (~8 px), a reading error of ±1 px corresponds to a relative error of approximately ±12% (1/8.06). Biological variability of the prism diameter was accounted for by considering a plausible range of 4–6 µm around 5 µm (±20%). Assuming independence of these two sources, the combined relative uncertainty is approximately √(0.12^2^ + 0.20^2^) ≈ 0.23–0.25; therefore, the scale is reported as estimated with approximately ±25% uncertainty. Under the 5 µm assumption, the working conversion is approximately 1.61 px/µm, so a 200 µm scale bar corresponds to approximately 322 px in the final figure.

The large language models artificial intelligence system ChatGPT5 (OpenAI) was used in the review phase to identify linguistic errors and check grammatical and typographical errors—typos and spacing

#### 2.7.1. Structure-Tensor Prism Orientation Mapping (Optical Proxy, Figure 3)

Input and ROI: Panel A is a cropped reflected-light micrograph showing enamel prism heads ('honeycomb' pattern). The same field of view was used to generate Panel B.

Pre-processing: The image was converted to greyscale (0–255) without contrast stretching or sharpening.

Local gradients: Spatial derivatives were computed with a 3 × 3 Sobel operator to obtain I*x* and I*y*.

Structure tensor and integration: For each pixel we formed the structure tensor as follows:

  $$\begin{array}{c} J=\left[\begin{array}{cc} I_{x}\;I_{y}\\ I_{x}\;I_{y} & {I_{y}}^{2} \end{array}\right]. \end{array}$$

We then smoothed *J* with a Gaussian integration window (*σ_i_* ≈ 3 px, kernel 13 × 13) to stabilise local orientation estimates.

Orientation and coherence: The in-plane orientation was computed as follows:

  $$\begin{array}{c} \theta =\frac{1}{2}\;a\;\tan\;2\left(\;2J_{xy},\;J_{xx}-J_{yy}\right)\;\left(\mathrm{mod}\;180{}^{\circ}\right). \end{array}$$

Anisotropy (‘coherence') was computed from the eigenvalues as follows:

  $$\begin{array}{c} C=\frac{\lambda_{1}-\lambda_{2}}{\lambda_{1}+\lambda_{2}},\text{ with }\lambda_{1}\geq \lambda_{2}\;\left(\text{range }0\text{–}1\right). \end{array}$$

Colour encoding and overlay: Orientation (*θ*) was mapped to hue; coherence (*C*) to saturation (low-coherence areas appear desaturated); the original normalised intensity was used as value. The HSV image was converted to RGB and blended with the original prism crop (*α* = 0.5) to produce Panel B. No histogram equalisation, denoising or edge-enhancement was applied beyond the tensor integration described above.

Software/reproducibility: Processing was implemented in Python (NumPy/SciPy/Pillow) using the standard structure-tensor pipeline; an equivalent workflow is available in FIJI/ImageJ (OrientationJ) with the same settings (Sobel gradients; Gaussian integration window *σ* ≈ 3 px; hue = orientation; saturation = coherence; value = original intensity).

Scale bar: The scale shown in the two-panel figure was added after generating Panel B by calibrating pixel size from five user-drawn prism-diameter markers in Panel A (median 16.76 px per prism; prism diameter assumed 5 µm → 0.298 µm/px). Because prism diameter can vary (≈4–6 µm) and the markers are short, we report the scale as estimated (overall uncertainty ~±20–25%).

## 3. Results

### 3.1. Selection of Sources of Evidence

**Figure 4:**
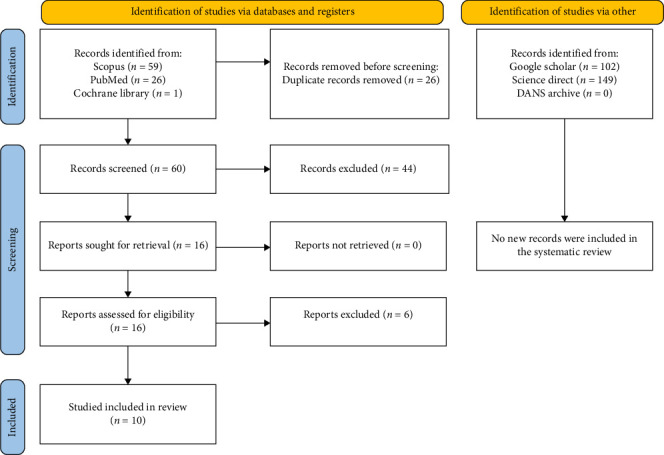
The entire selection and screening procedures are described in the PRISMA flowchart.

Searches conducted in ScienceDirect, SCOPUS, PubMed and the Cochrane Library yielded a total of 86 bibliographic sources. After removing duplicates, 60 unique sources remained. Among these, 16 articles were deemed potentially eligible, but only 10 fully met the eligibility criteria. Additionally, searches in grey literature sources (http://www.opengrey.eu, consulted on 7 March 2025; DANS EASY Archive; and Google Scholar) and previous systematic reviews did not identify any further studies for qualitative assessment (Figure 4). The entire process of study identification, selection and inclusion is outlined in the flow diagram illustrated in Figure 4.

The *k*-agreement between the two reviewers who performed the study selection was calculated, and the result was 0.65 (substantial agreement). The *k*-agreement was based on the formulas in the Cochrane Handbook for Systematic Reviews [30]. All data related to the measurement of the agreement have been reported in Table 1.

For greater clarity, a table has been provided and added, listing the web addresses of the databases, the date of the last search and the records identified, including grey literature (Table 2). The excluded reports with the relative reasons have been reported in Table 3.

### 3.2. Excluded Reports

The discussion of the main reasons for exclusion is detailed below.

The data reported by Hogg and Richardson [34] were excluded because the study results refer to mammals, specifically primates, canids, Artiodactyla and Proboscidea. During the initial selection phase of potentially eligible articles, this study was not excluded because the abstract did not explicitly mention analyses conducted on animals. However, this study demonstrated that the image compression ratio (ICR) method effectively distinguishes simple (radial) enamel from complex (decussated) enamel. Notably, samples with HSB (i.e., complex decussation) showed significantly higher ICR values than those with radial enamel [34]. Additionally, as supporting information, the study reported an ICR value of 0.0420 for *Homo sapiens* enamel.

Chen et al. [35] was erroneously included among the potentially eligible studies, as it is a literature review. This was not immediately evident from the title, although the abstract did contain this information. The review focuses on the biomechanical properties of cracks and fractures in teeth and their clinical implications, without specifically discussing HSB [37]. Instead, it addresses crack initiation, propagation and toughening mechanisms in dentine [35].

The article published by Roa and Ponce [36] is a terminological proposal related to HSB. Since neither the title nor the abstract contained clear elements indicating certain exclusion from the potentially eligible studies, a full-text analysis was performed. It became evident that the study did not provide any new results for discussion and was thus excluded, despite not being a literature review. However, this study highlights that the term ‘HSB' is an eponym that, while widely used, does not comprehensively describe the morphological characteristics of these structures. The authors propose replacing the eponymous term with two new ones: ‘diazone prismatica' and ‘parazone prismatica', aiming to unify terminology within the morphological research community [36].

The manuscript by Yilmaz et al. [33] was mistakenly included among the potentially eligible studies because the title did not indicate that it was a review. However, it was later excluded. The manuscript emphasises the hierarchical organisation of enamel, including structures such as HSB, which are fundamental to its fracture behaviour [33].

The study conducted by Chai [32] only partially focuses on HSB, and in a marginal manner. The analysis was performed using finite element modelling, and the manuscript mentions that HSB may enhance fracture resistance, as their organisation helps distribute and mitigate stresses, facilitating a ‘collaborative cracking' mechanism that, together with enamel tufts, provides stress shielding against crack propagation [32]. However, it does not contribute additional relevant information.

The manuscript published by Cantín and Fonseca [31] is a case report, and for this reason, it was excluded. The case report describes how HSBs are smaller and sparser on the external enamel surface of the mesiodens compared to the internal surface of invaginations [31].

### 3.3. Reports Included

A total of 10 articles were included in this scoping review: Desoutter et al. [38], Arrieta et al. [39], Arrieta and Line, [40], Harper et al. [41], Kienle and Schäfer, [42], Yang et al. [22], Collart-Dutilleul et al. [43], Grisimov [44] and Hegedűs et al. [45].

The extracted data were presented in two tables.  Table 4 summarises the first author, year of publication, country of origin, number and type of samples analysed and the type of analysis performed.  Table 5 includes, in addition to article-related information, key findings and novel contributions that the studies provide to the understanding, future applications and clinical implications of HSB.

Not all studies precisely reported the number of samples analysed. In particular, Grisimov [44] and Kienle and Schäfer [42], did not clearly state the number of teeth or sections examined, with Grisimov possibly analysing 48 sections. Hegedűs et al. [45] reported investigations performed on a single tooth. In the remaining seven studies, the total number of teeth examined was 288.

The analyses conducted ranged from optical microscopy and SEM to diffraction studies, histological investigations, microradiography and imaging analysis in the biometric field.

### 3.4. Risk of Bias

The risk of bias was assessed according to the guidelines provided in the CRIS [28], which are recommended for evaluating in vitro dental studies. The results are presented in Table 6, where each column was assigned a score from one to five (with 1 = low quality and 5 = high quality). The questions to which the reviewers responded by assigning scores were as follows:

• Sample size calculation: ‘Is the sample size adequate to achieve statistically significant results'?
• Significant difference between groups: ‘Was the measurement of the ‘significant difference' correctly set within the groups, considering the sample size and type of measurement'?
• Sample preparation and handling: ‘Does the study provide information on the production or handling of the samples to be tested'?
• Allocation sequence, randomisation and blinding: ‘Did the samples have an equal and independent chance of being assigned to any group'?
• Statistical analysis: ‘Are the statistical methods described'?

Across the 10 included in vitro studies, reporting quality and internal validity were heterogeneous. Sample-size justification was frequently absent or only partially addressed, and allocation/randomisation/blinding procedures were generally limited, resulting in variable scores for these domains (typically 2–4 for sample size; ≈3 for allocation/randomisation). By contrast, sample preparation/handling and statistical analysis were usually well described and consistently scored high. Several reports also lacked complete clarity on the exact number of teeth/sections analysed, which further constrains precision and external validity. Taken together, these features indicate that the current evidence base should be considered hypothesis-generating rather than practice-changing. Future studies would benefit from a priori sample-size calculations, transparent randomisation/blinding, prespecified analyses and full reporting per CRIS to reduce bias and improve reproducibility.

## 4. Discussion

The results of this scoping review highlight the crucial role of HSBs in enamel biomechanics, particularly in stress dissipation and crack propagation prevention. The results confirm that HSB organisation varies in different regions of the teeth, with a higher density in functional cusps and a lower density in cervical areas, influencing the mechanical strength and wear patterns of enamel. Furthermore, recent advances in imaging technologies, such as SEM, Raman microscopy and coherent light scattering, have provided new insights into the structural and chemical properties of HSBs, suggesting potential diagnostic and restorative applications. Despite these advances, several gaps in current knowledge remain, particularly regarding the clinical implications of HSBs in relation to non-carious cervical lesions (NCCLs), enamel abfraction and demineralisation. The following discussion will critically review these aspects, comparing the current results with existing literature, identifying limitations and outlining future research directions.

### 4.1. HSB and Their Role in Abrasion and NCCLs

The presence of abrasions and fissures in the enamel structure leads to a reduction in the resilience capacity of the crystalline structure. In this context, the HSB play a role in arresting cracks in the enamel [47]. The intertwined arrangement of prisms determines the end of crack progression by radiating the fissure [32].

The orientation of the prisms can affect abrasion differently. Prisms with a long axis parallel to the occlusal surface tend to undergo a more intense abrasive process. In fact, when enamel prisms intersect the external surface at a right angle, the structure becomes more resistant [48]. The angle at which each individual prism interfaces with the tooth's surface is crucial for its wear resistance [49].

Throughout evolution, prisms have optimally adapted to resist abrasion through an asymmetrical configuration within the enamel [50, 51]. Higher packing densities of HSB (reflecting a more complex arrangement of individual prisms) occur in enamel areas more susceptible to abrasion, such as the occlusal contact areas of teeth, while cervical surfaces are relatively devoid of HSB [46]. This reduced density of HSB reflects enamel with less prism decussation [22], making the surface more susceptible to processes like erosion [46] and abrasion [52, 53].

Abrasive mechanisms primarily affecting the cervical surface, along with acidic tooth erosion, form the basis for NCCLs [54].

The most influential abrasive action in the development of NCCL in the last century has been the widespread and often improper use of industrial toothbrushes, leading to an increased incidence of NCCLs [55]. Another determining factor has been the change in dietary habits over recent centuries. The production of refined sugar has resulted in an increase in dental caries in the population [56], and the consumption of processed flours and foods has reduced occlusal surface abrasion, which was common in pre-industrial populations. In some ancient populations, abrasion was the main cause of tooth loss [57]. The reduction of occlusal table and decreased vertical crown dimension has led to occlusal forces having a reduced impact in NCCLs formation, as defined by some authors as abfraction. This partly explains the absence of wedge-shaped lesions in ancient populations, where there were no brushing and occlusal abrasion traumas [58, 59].

The loss of dental enamel in cervical areas, according to the abfraction theory, is due to concentrated bending forces at the CEJ [60]. Teeth subjected to bending forces during occlusion experience tension and compression in opposite parts of the dental element, leading to enamel loss and wedge-shaped lesions in the area where these forces converge [61]. Cyclic loading causes the formation of fissures and fractures that separate enamel prisms, allowing small molecules and water to penetrate between the prisms, preventing the restoration of interprismatic bonds once the force application ceases.

However, there is no unanimous consensus among dentists regarding the possibility that occlusal load is the sole aetiological factor responsible for ‘abfraction' lesions [62]. Instead, a multifactorial aetiology is suggested, in which erosive phenomena (such as the action of acidic foods) and abrasive factors (excessive brushing) contribute alongside abnormal occlusal and non-axial loads to the formation of wedge-shaped lesions on the vestibular cervical surface [63]. In fact, there is a lack of randomised clinical studies demonstrating that abnormal occlusal loads alone can cause NCCLs [64], except for in vitro or laboratory studies [65].

In a previous literature review, Lynch et al. [47] investigated clinical aspects related to HSBs, concluding that the way they are distributed appears to passively facilitate conditions like abfraction and the syndrome of fractured teeth [65, 66].

The authors suggest that occlusal forces now play a greater role in the aetiology of NCCLs. This is due to the reduced abrasion of the occlusal surface and vertical dimension of the crown observed in modern populations, which contrasts with ancient populations where occlusal abrasion was more evident. Consequently, the impact of occlusal forces on the formation of NCCL, often referred to as abfraction, has increased [60]. This increased role of occlusal forces partly explains why wedge-shaped lesions were absent in ancient populations, where neither toothbrushing nor occlusal abrasion trauma were factors.

In the cervical zones of teeth [67], the longitudinal orientation of prisms with respect to the external surface and the reduced density of HSB [52] predispose the area to erosive (mainly due to a carbohydrate-rich and acidic diet) and abrasive (excessive or incorrect tooth brushing) phenomena [68–70]; however, the enamel striae reported by Sivasithamparam et al. [69] could represent superficial perichymata. Considering that the current population retains the occlusal surface and crown vertical dimension [71], occlusal forces can exert bending and compression forces, which, in combination with these two mechanisms, can contribute to the characteristics of lesions described by many authors as abfraction [72].

From a conservative dentistry perspective, it's important to note that, especially in anterior teeth, tooth characterisation is based on mimicking optical features of both the surface and deeper regions of enamel and partly dentine. HSB can be visualised without the need for tooth sectioning, for example, through photographic images with macro lenses, and their visualisation has even been considered a biometric system for personal identification in automated systems [73].

**Figure 5:**
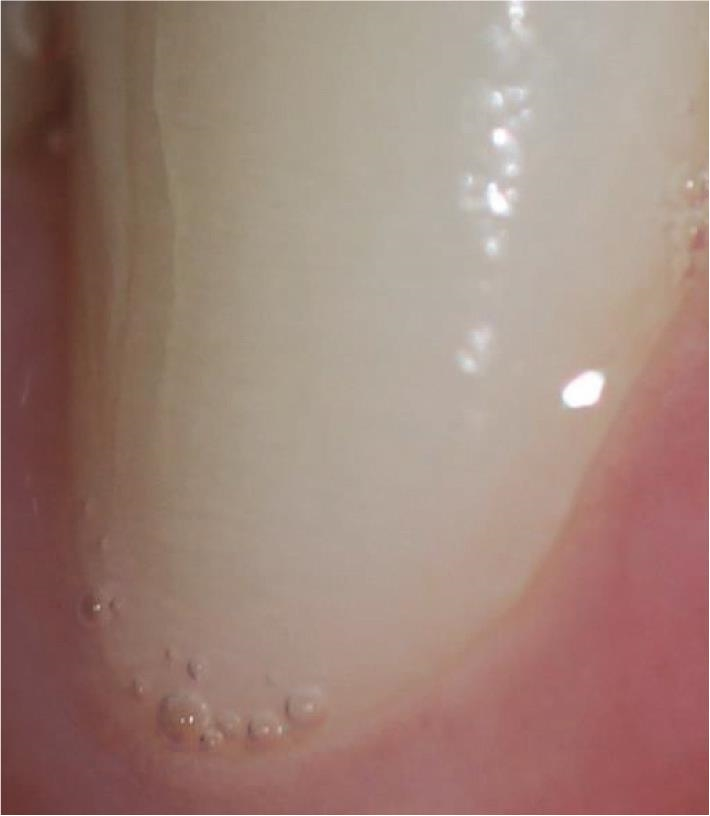
Lower canine: visualisation of a weak perikymata arranged perpendicularly to the long axis of the tooth determined by the decussation of the enamel prisms attributable to the Hunter–Schreger bands.

**Figure 6:**
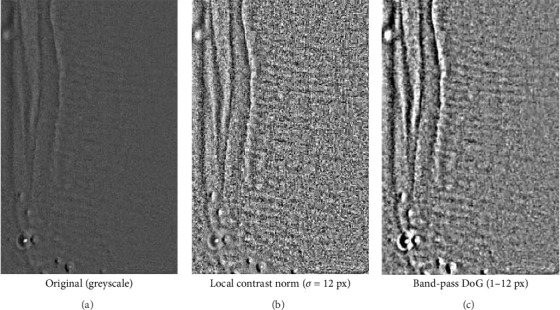
Clinical enamel close-up and contrast-enhanced views. (A) Greyscale image. (B) Local contrast normalisation (Gaussian *σ* = 12 px). (C) Band-pass filtering (difference-of-Gaussians, 1–12 px). Enhancements are used solely to improve visualisation of surface refractive banding compatible with HSB; they provide an optical texture proxy for local prism-bundle orientation and are not a direct crystallographic measurement.

**Figure 7:**
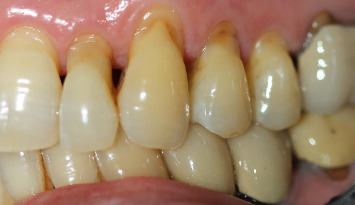
NCCL affecting the teeth: incisors, canines and upper left premolars.

Visualising HSB through external tooth observation could have clinical relevance in conservative dentistry, especially in the pre-reconstruction phase. It is believed that HSB are responsible for the internally visible perichemate [70], while the perikemes that are manifested with striated alterations on the external surface are considered expressions of the striae of Retzius [74] (Figures 5, 6 and 7).

For clarity, building on Figure 5—which shows a macro-detail of the buccal enamel surface with reflectance variations compatible with HSB—we present a clinical close-up acquired at moderate resolution (Figure 6). Part A of Figure 6 shows the image in greyscale; parts B and C apply low-intensity contrast enhancement (local contrast normalisation) and a gentle band-pass filter (difference-of-Gaussians). These steps, previously used to improve the readability of HSB on intact teeth, highlight parallel, weakly periodic striae that become easier for the reader to discern, without introducing geometric artefacts (the filters are linear and do not deform morphology).

Consistent with the optical model of HSB, the light–dark alternations reflect differences in the orientation of prism bundles (parazones/diazones) relative to illumination and viewing geometry. We emphasise that this is an optical visualisation of surface texture—a proxy for local prism-bundle orientation—not a direct crystallographic measurement; the latter requires techniques such as SEM/EBSD or polarised-light microscopy on thin sections. The main limitations of this surface visualisation are its sensitivity to illumination, surface curvature and image optical quality; to minimise over-processing we avoided aggressive histogram equalisation and non-linear sharpening. The visual evidence in Figure 6 therefore complements the previous clinical images and, at a sub-macroscopic scale, makes explicit the refractive variations expected from alternating prism orientations.

### 4.2. Literature Analysis From the Last 15 Years

The study conducted by Desoutter et al. [38] on 10 human mandibular molars, using microtomography, indicated that the formation of enamel tufts is linked to HSB, as the mechanical properties of enamel are influenced by the presence of enamel tufts and their correlation with variations in HSB orientation. These findings are further supported by the HSB analysis performed by Yang et al. [22] on 41 molars, demonstrating that the density and packing of HSB, determined by prism decussation, help to dissipate energy and redirect crack propagation, serving as a damage control mechanism during the mastication cycle. The study shows that functional cusps exhibit a higher HSB density compared to guiding cusps. These structural differences suggest that HSB contribute to strengthening functional cusps against fractures, whereas guiding cusps, with less decussation, are inherently more susceptible to crack propagation and chipping [22].

Furthermore, a Raman microscopy study conducted by Collart-Dutilleul et al. [43] on 20 extracted teeth observed variations in hydroxyapatite concentration among HSB, enabling a comparison of filling rates between different HSB orientations. A greater HSB packing, as highlighted by Lynch et al. [46], is correlated with an increase in prism decussation points, which act as an anti-fracture mechanism, preventing crack propagation and improving abrasion resistance. The asymmetric distribution of HSB, with a higher concentration in areas subjected to high masticatory loads, aligns with subsequent findings by Yang et al. [22], indicating minimal HSB densities in cervical regions [75], which gradually increase towards high-load areas such as the occlusal surfaces of posterior teeth and incisal edges of anterior teeth.

These studies further support the notion that anomalies in HSB formation or arrangement could indicate a greater predisposition to fractures or lesions, such as abfraction. This suggests that HSB contribute to optimising the mechanical behaviour of enamel. These insights also align with the hypothesis described in the systematic review by Yilmaz et al. [33], in which HSB are described as a crucial component of enamel's hierarchical structure. This organisation allows for the deflection and deceleration of crack propagation, contributing to the R-curve effect, which enhances fracture resistance as crack length increases. In other words, HSB function as toughening mechanisms [35], where crack interfaces between bands with different orientations force cracks to deviate from their trajectory, slowing propagation. This facilitates processes such as crack bridging and crack path deflection, thereby increasing enamel's damage tolerance, albeit with a trade-off in ultimate fracture strength compared to a fully homogeneous material.

The chemical properties of enamel also appear to be influenced by HSB orientation. A study by Harper et al. [41] observed that lactic acid demineralization, visualised using polarised light imaging, follows HSB patterns, with certain areas being more susceptible to demineralization. This insight could guide the development of targeted preventive strategies, such as specific demineralising treatments [41].

Enamel mineralisation has also been investigated through coherent light diffraction analysis conducted by Grisimov [44], which indicates that as patients age, enamel mineralisation tends to reduce optical anisotropy, leading to a decrease in HSB contrast.

The advancement of imaging and microscopy technologies has further expanded research possibilities in the field of HSB. The study by Hegedűs et al. [45], using SEM analysis and image-processing software, suggests potential applications in dental research and restorative treatments. Colour-coded imaging techniques provide valuable insights for optimising adhesive strategies by identifying areas where prism orientation, expressed through HSB, enhances enamel adhesion and mechanical resistance [45].

Future applications of HSBs may also extend into forensic dentistry for personal identification [76]. The software used to visualise HSB is constantly being updated to compensate for the low contrast between light and dark bands visible on the external tooth surface. Arrieta et al. [39] have developed a computationally efficient method [40] that significantly improves contrast and minimises intensity variations in HSB imaging [39]. Additionally, some studies have successfully simulated HSB optical behaviour using the Monte Carlo method. OCT imaging has proven to be a valuable diagnostic tool for the early detection of structural alterations in enamel [42].

Collectively, these studies highlight that human enamel is a highly complex and anisotropic material. Its hierarchical structure, from nanoscale hydroxyapatite crystallites to microscale enamel prisms, influences both its optical properties and mechanical resilience. The greater decussation of enamel prisms in functional areas of molars likely serves as an intrinsic toughening mechanism, helping dissipate stress and resist fractures. Advanced imaging techniques such as OCT, confocal Raman microscopy and diffraction analysis not only enhance our understanding of these structural characteristics but also offer powerful tools for applications in dental diagnostics, biomimetics, forensic science and evolutionary anthropology.

Beyond forensic applications, in vivo HSB mapping may inform paediatric and restorative care by: (i) identifying cusps at higher risk of craze-line/crack progression, (ii) supporting HSB-aware bevel/sealant planning to improve retention and marginal integrity and (iii) exploring associations between HSB orientation/spacing and site-specific erosive/attritional wear. We therefore include a focused in vivo research agenda (Table 6) specifying clinical questions, designs and endpoints.

These findings, derived from morphological, mechanical and spectroscopic studies, underscore the fundamental role of HSB organisation in ensuring the structural and functional integrity of dental enamel.

### 4.3. Limitations of the Review and Future Studies

The main limitations of this review stem from the lack of studies correlating clinical conditions with HSB. Current dental and medical knowledge provides only indirect evidence on how HSB might influence the onset of abfraction lesions (noting that this remains a theoretical hypothesis, on which the authors take a neutral stance) or NCCLs.

Despite advancements, our understanding of the relationship between HSB and dental pathologies, as well as the progression of the carious process, remains incomplete. In vitro data are valuable but do not always accurately reflect the physiological conditions of a living tooth, which is continuously exposed to occlusion cycles, erosion and abrasion.

Most included studies are in vitro, often with limited sample-size justification and randomisation; accordingly, findings should be interpreted as hypothesis-generating rather than practice-changing.

The inclusion of only 10 in vitro studies conducted over the past 15 years, covering a total of 288 teeth, may limit the generalizability of our conclusions.

Future research should prioritise clinical trials [77], preferably incorporating advanced imaging techniques that enable in vivo observation of HSB (as proposed by Arrieta et al. [39]). In contrast to in vitro studies, longitudinal investigations could monitor the progression of lesions over time, such as carious and non-carious lesions, to gain a clearer understanding of their development dynamics. Additionally, research should explore how adhesive and restorative materials interact with varying HSB distributions, which could significantly influence clinical outcomes in restorative dentistry.

In addition, in vivo studies of HSB and its mapping may have clinically relevant implications. In vivo HSB mapping—even using optical proxies such as cross-polarised macro-photography coupled with orientation/coherence analysis—could inform several clinical decisions. The superficial light–dark banding visible on enamel (HSB; Figure 6) reflects changes in prism-bundle orientation that may influence crack trajectories and local enamel fracture resistance; moreover, band orientation appears to co-vary with patterns of erosive/attritional wear, as discussed in relation to the origins of NCCLs. Accordingly, where banding is visible, chairside in vivo HSB mapping using texture-based optical measurements (a proxy for local prism orientation) could be implemented in a routine dental setting, providing an additional tool to identify cusps at higher risk of craze-line progression and to guide restorative planning or preventive cuspal coverage.

Three concrete in vivo research questions are (Table 7) as follows:

1. Do teeth with ‘unfavourable' local HSB orientation show faster growth of fracture lines/craze-lines?
2. Is HSB orientation/spacing associated with site-specific erosive or attritional wear?
3. Do bevels and sealants placed with respect to local HSB morphology improve retention and marginal integrity compared with control?

## 5. Conclusion

In conclusion, a comprehensive analysis of the literature clearly demonstrates that HSB represent a crucial structural component of enamel, whose evolutionary and functional organisation plays a significant role in energy dissipation and crack prevention, thereby enhancing dental resistance. It is therefore highly desirable to develop additional investigative methodologies, including longitudinal clinical studies and in vivo trials, to confirm the role of HSB in preventing dental lesions and to drive innovation in restorative treatments.

## Figures and Tables

**Table 1:** *k*-Agreement calculation.

$k\;=\frac{\left(P_{o}-\;P_{e}\right)}{\left(1-P_{e}\right)}$					
Reviewer	Decision	Reviewer 2	Reviewer 2	Reviewer 2	
		Include	Unsure	Exclude	Total
Reviewer 1	Include	9	1	0	10
Reviewer 1	Unsure	0	0	1	1
Reviewer 1	Exclude	0	1	4	5
	Total	9	2	5	16

*Note:* Proportion of agreement, Po = 0.81; agreement expected, Pe = 0.46; *k*-agreement = 0.65 (<0 no agreement, 0.0–0.20 slight agreement, 0.21–0.40 fair agreement, 0.41–0.60 moderate agreement, 0.61–0.80 substantial agreement and 0.81–1.00 almost perfect agreement).

**Table 2:** Table showing the addresses of the databases where the records were searched.

Research databases or bibliographic databases	Web address	Data	Records
PubMed	https://pubmed.ncbi.nlm.nih.gov/?term=Hunter%E2%80%93Schreger+bands&filter=years.2010-2025&sort=date&size=200	7 March 2025	26
	Search: Hunter–Schreger bands Filters: from 2010 to 2025 Sort by: Most Recent (“Hunter-Schreger”[All Fields] AND (“band s”[All Fields] OR “bands”[All Fields])) AND (2010 : 2025[pdat]) Translations bands: “band's”[All Fields] OR “bands”[All Fields]		

Scopus^a^	https://www.scopus.com/results/results.uri?st1=hunter%E2%80%93schreger+AND+bands&st2=&s=TITLE-ABS-KEY%28hunter%E2%80%93schreger+AND+bands%29&limit=10&origin=resultslist&sort=plf-f&src=s&sot=b&sdt=cl&sessionSearchId=4ed4dddc48c6d24767ef5eb790dde8dc&yearFrom=2010&yearTo=2025	7 March 2025	59
	TITLE-ABS-KEY (hunter–schreger AND bands) AND PUBYEAR > 2009 AND PUBYEAR < 2026		

Cochrane Central Register of Controlled Trials (CENTRAL)	https://www.cochranelibrary.com/search	7 March 2025	1
	Hunter–Schreger bands in Title Abstract Keyword		

Google Scholar^a^	https://scholar.google.com/scholar?q=Hunter%E2%80%93Schreger+bands&hl=it&as_sdt=0,5&as_ylo=2010&as_yhi=2025&as_rr=1	7 March 2025	102
	Hunter–Schreger bands		

ScienceDirect^a^	https://www.sciencedirect.com/search?qs=Hunter%E2%80%93Schreger%20bands%20&years=2010%2C2011%2C2025%2C2024%2C2023%2C2022%2C2021%2C2020%2C2019%2C2018%2C2017%2C2016%2C2015%2C2014%2C2012%2C2013%2C2009	7 March 2025	1491

^a^You must be logged in to Scopus, ScienceDirect and Google Scholar.

**Table 3:** Excluded reports.

Autor, data, reference	Country	Reason for exclusion
Cantín and Fonseca, 2013 [31]	Argentina	Case report
Chai, 2022 [32]	Israel	Finite element analysis not involving HSB
Yilmaz et al., 2015 [33]	Germany and Australia	Literature review
Hogg and Richardson, 2019 [34]	USA	Study conducted on animal teeth
Chen et al., 2023 [35]	China, USA and Italy	Literature review
Roa and Ponce, 2019 [36]	Chile	Terminology proposal

**Table 4:** Data extraction table.

Autor, data	Country	Type and number of samples	Imaging/analysis technique
Hegedűs et al., 2024 [45]	Hungary	1 Molar tooth	SEM
Desoutter et al., 2022 [38]	France	10 Mandibular teeth	Microtomography
Arrieta et al., 2018 [39]	Brazil	36 Mandibular incisor teeth	Digital image analysis, ImageMagick
Arrieta and Line, 2017 [40]	Brazil	11 Mandibular incisor teeth	Digital image analysis, ImageMagick
Harper et al., 2021 [41]	UK and Italy	25 Sections taken from 10 human third molars	Microradiography
Kienle and Schäfer, 2012 [42]	Germany	Sections of extracted human teeth	OCT integrated by Monte Carlo simulations
Yang et al., 2022 [22]	United States	41 Human molars (21 maxillary 20 mandibular)	SEM
Collart-Dutilleul et al., 2025 [43]	France and Argentina	20 Extracted teeth	Raman confocal microscopy
Grisimov, 2020 [44]	Russia	≈46 levels per tooth	Diffraction of coherent light beams
Lynch et al., 2010 [46]	UK	160 Teeth	Reflected light microscopy

Abbreviations: OCT, optical coherence tomography; SEM, scanning electron microscopy.

**Table 5:** Data extraction table (main results and future implication).

Autor, data	Main findings regarding HSB
Hegedűs et al., 2024 [45]	Quantification of the orientation of enamel prisms revealing that HSB are characterised by systematic variations in azimuthal angles that precisely define their boundaries and width along the enamel.
Desoutter et al., 2022 [38]	HSB appear to arise from variations in the regular undulation of tuft drapes.
Arrieta et al., 2018 [39]	Significantly increases the contrast and uniformity of Hunter–Schreger band images in intact teeth, expanding the area and number of detectable bands and facilitating automated analysis of enamel microstructure.
Arrieta and Line, 2017 [40]	Image optimisation with a polarising filter together with corn oil treatment with significantly increased contrast and the observable area of HSB.
Harper et al., 2021 [41]	The study highlighted that the anisotropic orientation of hydroxylapatite crystallites, organised in Hunter–Schreger bands, drives the preferential progression of enamel demineralisation.
Kienle and Schäfer, 2012 [42]	The study demonstrates that Hunter–Schreger bands arise from the sinusoidal oscillation of enamel prisms and can be accurately reproduced by Monte Carlo simulations based on solutions of Maxwell's equations.
Yang et al., 2022 [22]	The main conclusions of the study indicate that ‘functional' cusps of human molars show a higher HSB density and a higher relative thickness of decussate enamel than ‘guide' cusps.
Collart-Dutilleul et al., 2025 [43]	Variations in the hydroxyapatite concentration were observed between the HSB with the possibility of comparing the filling rates between the two HSB orientations.
Grisimov, 2020 [44]	The presence or absence of a diffraction pattern corresponding to diffraction on periodic inhomogeneities of tooth enamel depends on the age of the tooth and the thickness of the section.

**Table 6:** Risk of bias.

First author, data	Sample size calculation	Meaningful difference between groups	Sample preparation and handling	Allocation sequence, randomisation and blinding	Statistical analysis	Score
Hegedűs et al., 2024 [45]	2	2	5	3	5	17
Desoutter et al., 2022 [38]	4	4	5	3	5	21
Arrieta et al., 2018 [39]	5	3	5	3	3	19
Arrieta and Line, 2017 [40]	5	3	5	3	3	19
Harper et al., 2021 [41]	5	5	5	3	5	23
Kienle and Schäfer, 2012 [42]	2	3	5	3	3	16
Yang et al., 2022 [22]	4	5	5	3	5	22
Collart-Dutilleul et al., 2025 [43]	5	5	5	3	5	23
Grisimov, 2020 [44]	3	3	5	3	3	17
Lynch et al., 2010 [46]	4	4	5	3	5	21

**Table 7:** Clinical translation of HSB: proposed in vivo studies.

Clinical question	Proposed design	Primary endpoints	Instrumentation/methods
1. Crack/craze-lines risk-do teeth with ‘unfavourable' local HSB orientation show faster growth of fracture lines/craze-lines?	Prospective cohort; baseline HSB map (cross-polarised macro → orientation/coherence); 6–12-month follow-up	Change in length/number of lines; symptoms; need for cuspal coverage; fracture events	Standardised macro-photography; HSB mapping structure-tensor analysis (orientation/coherence); FOTI/transillumination; serial photography; control of confounders (age, bruxism and restorations)
2. Site-specific wear/erosion-is HSB orientation/spacing associated with site-specific erosive or attritional wear?	Cross-sectional plus 1-year follow-up; correlation between HSB metrics (orientation and spacing) and wear patterns	Wear progression (3D profilometry), site localisation; sensitivity; clinical indices of erosion/attrition	Standardised macro-photography; HSB mapping; 3D profilometry; diet/habit questionnaires; multivariable analysis
3. HSB-guided bevel/sealant-do bevels/sealants aligned with local HSB patterns improve retention and marginal integrity versus control?	Split-mouth RCT; assessments at 6/12/24 months	Sealant loss; marginal staining/microleakage; repair rate; post-op sensitivity	Standardised macro-photography; HSB mapping, standardised adhesive protocols; operator checklist; intention-to-treat analysis

## Data Availability

Data sharing is not applicable to this article as no new data were created or analysed in this study.
